# Candidemia en Colombia

**DOI:** 10.7705/biomedica.4400

**Published:** 2020-03-30

**Authors:** Jorge Alberto Cortés, José Franklin Ruiz, Lizeth Natalia Melgarejo-Moreno, Elkin V. Lemos

**Affiliations:** 1 Departamento de Medicina Interna, Facultad de Medicina, Universidad Nacional de Colombia, Bogotá, D.C., Colombia Universidad Nacional de Colombia Departamento de Medicina Interna Facultad de Medicina Universidad Nacional de Colombia BogotáD.C Colombia; 2 Grupo de Investigación en Enfermedades Infecciosas, Facultad de Medicina, Universidad Nacional de Colombia, Bogotá, D.C., Colombia Universidad Nacional de Colombia Grupo de Investigación en Enfermedades Infecciosas Facultad de Medicina Universidad Nacional de Colombia BogotáD.C Colombia; 3 Departamento Médico, Pfizer, Bogotá, D.C., Colombia Departamento Médico BogotáD.C Colombia

**Keywords:** candidiasis, candidemia, unidad de cuidados intensivos, micosis, infecciones fúngicas invasivas, candidiasis invasiva, Colombia, Candidiasis, candidemia, intensive care units, mycoses, invasive fungal infections, candidiasis, invasive, Colombia

## Abstract

En Colombia, especialmente en las unidades de cuidados intensivos, la candidemia es una causa frecuente de infección del torrente sanguíneo y representa el 88 % de las infecciones fúngicas en pacientes hospitalizados, con una mortalidad entre 36 y 78 %. Su incidencia en Colombia es mayor a la reportada en los países desarrollados e, incluso, en otros países de Latinoamérica. Para su manejo deben considerarse los factores de riesgo del paciente, luego valorar las características clínicas y, finalmente, hacer los estudios microbiológicos y, si es necesario, pruebas moleculares.

En general, las guías estadounidenses, latinoamericanas y europeas recomiendan las equinocandinas como el tratamiento de primera línea de la candidemia y difieren en el uso de fluconazol dependiendo de la 'evidencia', la gravedad de la enfermedad, la exposición previa a los azoles y la prevalencia de *Candida* no *albicans.* Dada su gran incidencia en nuestro país, asociada con una elevada mortalidad, esta infección debe buscarse sistemáticamente en pacientes con factores de riesgo, con el fin de iniciar oportunamente el tratamiento antifúngico.

## Abstract (en)

In Colombia, especially in intensive care units, candidemia is a frequent cause of infection, accounting for 88% of fungal infections in hospitalized patients, with mortality ranging from 36% to 78%. Its incidence in Colombia is higher than that reported in developed countries and even higher than in other Latin American countries. First, the patient's risk factors should be considered, and then clinical characteristics should be assessed. Finally, microbiological studies are recommended and if the evidence supports its use, molecular testing. In general, American, Latin American, and European guides place the echinocandins as the first-line treatment for candidemia and differ in the use of fluconazole based on evidence, disease severity, previous exposure to azoles, and prevalence of *Candida non-albicans.* Taking into account the high incidence of this disease in our setting, it should be looked for in patients with risk factors to start a prompt empirical anti-fungal treatment.

El aumento de la población de adultos mayores, pacientes oncológicos, con trasplante e inmunocomprometidos, implica un crecimiento significativo de la población en riesgo para el desarrollo de complicaciones infecciosas, entre ellas la candidemia, cuya incidencia aumenta de manera notoria en la población críticamente enferma. Hasta el 85 % de las infecciones fúngicas en las unidades de cuidados intensivos corresponde a infecciones por *Candida* spp. ^1^ y su mortalidad es cercana al 40 % ^2^. Por otro lado, está la resistencia a los antifúngicos disponibles para el tratamiento de la candidemia: en Bogotá se ha establecido la resistencia al fluconazol hasta en 30 % de los aislamientos ^1^ y, en Medellín, en el 22 % de los casos ^3^.

El objetivo de la presente revisión fue compilar la información disponible en los últimos 10 años sobre la candidemia en Colombia.

## Candidemia

La candidiasis, es decir, la infección producida por las levaduras del género *Candida,* comprende un espectro clínico que abarca infecciones superficiales y diseminadas que afectan cualquier órgano o sistema. Las especies de *Candida* son parte de la flora normal del cuerpo humano, pero solo un pequeño porcentaje de las especies identificadas causan enfermedad. *Candida albicans* es responsable de la mitad de los casos ^4^, aunque su frecuencia parece estar disminuyendo en los últimos años ^5^^,^^6^.

La candidiasis invasiva ocurre cuando las levaduras del género *Candida* alcanzan el torrente sanguíneo, desde donde pueden diseminarse a cualquier tejido de la anatomía humana. La candidiasis invasiva incluye un amplio espectro: candidemia con endoftalmitis o sin ella, infecciones hematógenas diseminadas, compromiso de un órgano (infecciones abdominales, peritonitis, meningitis y endocarditis) y candidiasis hepatoesplénica, principalmente en pacientes con enfermedad hematológica. La fuente de infección por *Candida* spp. puede ser endógena, de la flora gastrointestinal o de la colonización mucocutánea, o exógena, de las manos de los trabajadores de la salud o de objetos o infusiones contaminadas ^7^. La candidemia es la forma clínica de la candidiasis invasiva que ocurre más frecuentemente. La frecuencia de las diferentes especies de *Candida* en esta infección varía geográficamente y en el tiempo, como se explicará posteriormente.

## Factores de riesgo

El principal factor de riesgo es la gravedad de la condición de base del paciente, la cual usualmente se valora con la puntuación de la *Acute Physiology and Chronic Health Evaluation II* (APACHE II), cuyo uso ha sido validado en las unidades de cuidados intensivos, aunque en muchos estudios se ha usado para todos los pacientes.

La gravedad de la enfermedad implica factores adicionales de riesgo, como el uso de antibióticos de amplio espectro, las cirugías mayores, la nutrición parenteral total y el uso de dispositivos invasivos como sondas y catéteres ^8^. Otros factores de riesgo descritos son la duración de la estancia hospitalaria, la edad avanzada, la presencia de múltiples comorbilidades, la diabetes mellitus, los trasplantes, las enfermedades neoplásicas, la cirugía gastrointestinal, los implantes protésicos, los tratamientos inmunosupresores, la pancreatitis, la desnutrición, las quemaduras graves, el trauma, la colonización previa por *Candida* spp., la falla renal aguda -principalmente en pacientes con diálisis-, el compromiso del sistema inmunológico, el bajo peso al nacer, la neutropenia, la hospitalización en unidades de cuidados intensivos, la disfunción orgánica que requiere procedimientos invasivos como la asistencia respiratoria mecánica, los medicamentos vasoactivos, la diálisis, la circulación extracorpórea, las transfusiones y la traqueotomía, entre otros (cuadro 1) ^2^^,^^7^^,^^9^^,^^10^. Un estudio realizado en tres instituciones de diferentes partes de Colombia, validó la frecuencia e importancia de los factores de riesgo mencionados ^11^.

**Cuadro 1:** Factores de riesgo para candidemia en pacientes hospitalizados

Factor	Condicionantes
Tiempo de hospitalización	Exposición a múltiples factores de riesgo
Microbiota colonizadora
	Tratamiento con antibióticos
	Colonización por Candida
	Problemas de control de infecciones
Inmunosupresión
	Quimioterapia
	Corticoesteroides
	Cáncer
	Neutropenia
	Prematuridad
Procedimientos médicos invasivos
	Catéter intravascular
	Respiración mecánica asistida
	Cirugía
	Hemodiálisis
	Nutrición parenteral

Uno de los factores de riesgo más relevantes para las candidiasis invasivas es el uso de antimicrobianos. Los antibióticos tienen varios efectos que favorecen la multiplicación de las levaduras, entre ellos, la eliminación de la microbiota nativa que establece un control ecológico sobre las especies de *Candida* presentes en el tubo digestivo y la limitación de la diversidad de especies que se encuentran en un momento dado. Aunque algunos antibióticos se han asociado más que otros con infecciones por *Candida* spp. ^11^, cualquiera de amplio espectro puede tener este efecto.

## Fisiopatología

Las levaduras de *Candida* spp. viven normalmente en el cuerpo sin causar daño. Cuando hay compromiso de la inmunidad o una condición que conlleve el desequilibrio de la flora normal, la cantidad de levaduras aumenta produciendo su colonización excesiva y el paso al torrente sanguíneo mediante translocación ^9^. Los pacientes con cirugía abdominal y nutrición parenteral tienen mayor riesgo de infecciones de origen endógeno debido, en ambos casos, a la alteración de la fisiología gastrointestinal y la consecuente pérdida del epitelio o de sus uniones, lo cual favorece el paso desde la luz intestinal hasta los vasos sanguíneos. Lo mismo sucede en los pacientes con neutropenia posterior a la quimioterapia, la cual produce lesiones importantes del epitelio que se manifiestan como diarrea y mucositis, y se refleja en otros epitelios como el cuero cabelludo o las células hematopoyéticas ^9^.

Por otro lado, las fuentes de infección exógena incluyen la contaminación de las manos del personal de atención, de las soluciones parenterales y oftálmicas, y de dispositivos como catéteres, válvulas cardíacas y respiradores, entre otros ^12^.

En un estudio en unidades neonatales de cuidados intensivos de Bogotá, se evidenció la frecuencia elevada de infección ocasionada por el mismo tipo de especies de *Candida* identificadas como colonizadoras en los pacientes ^13^, así como el de un brote relacionado con problemas de control de infecciones en estas unidades, en las que se comprobó la colonización de catéteres centrales y la posterior infección debido a deficientes medidas de limpieza y desinfección de los puertos endovenosos ^14^. Además, se ha estimado que, en condiciones de cuidados intensivos, más del 30 % de los casos observados estarían relacionados con brotes ^15^.

Entre los factores de virulencia que determinan la relación de *Candida* spp. con el huésped, se ha demostrado su capacidad de adherirse a los tejidos y a las superficies de catéteres o prótesis, lo que les permite colonizar y formar una biopelícula que limita la acción de los antimicrobianos. La biopelícula frena el metabolismo y el crecimiento del hongo, lo cual disminuye los sitios de acción de la mayoría de los antifúngicos ^9^. Asimismo, se ha establecido que las biopelículas ayudan al incremento de los genes de resistencia de las levaduras ^16^. Otros factores determinantes de la diseminación e invasión de los tejidos son la producción de enzimas (proteasas, fosfolipasas y lipasas) y el cambio morfológico de *Candida* spp. de blastoconidia o seudohifa a hifa ^12^.

## Epidemiología en Colombia

La frecuencia de la candidemia varía según los servicios de hospitalización y los factores de riesgo de los pacientes. Además, se han observado cambios en la epidemiología de las especies de *Candida* en cuanto a la incidencia y la resistencia a los antifúngicos por área geográfica ^12^. En un estudio epidemiológico de siete países latinoamericanos que incluía pacientes colombianos, se reportó una incidencia general de candidemia de 1,18 casos por cada 1.000 admisiones en el conjunto de hospitales incluidos en el estudio.

En Colombia se reportó la incidencia más alta: 1,98 casos y, en Chile, la más baja: 0,33 casos por 1.000 admisiones ^5^. Estos datos contrastan con los de tasas más bajas de incidencia de candidemia reportadas en los Estados Unidos, con 0,96 casos, y en Europa, con 0,38 casos por 1.000 admisiones ^2^.

En un estudio de siete hospitales de Colombia entre el 2004 y el 2008, se reportó que la frecuencia de candidiasis invasiva era superior a la establecida en países desarrollados. La incidencia fue de 2,3 casos por 1.000 días de estancia en una unidad de cuidados intensivos, con una incidencia global observada de 1,4 % por egresos de la unidad ^17^. Se observó una tendencia al aumento de la incidencia y la prevalencia de candidemia, principalmente por especies de *Candida* no *albicans,* las cuales correspondieron a 44 % de los aislamientos.

Esta dinámica epidemiológica tiene implicaciones importantes en el tratamiento, dada la menor sensibilidad de estas especies al fluconazol ^17^. Las razones de los cambios de la incidencia, así como de los cambios epidemiológicos observados en Colombia, no son claras y ameritan estudios apropiados para entender su dinámica. Según un estudio de la Corporación para Investigaciones Biológicas (CIB) de Medellín, la candidiasis invasiva representaba el 75 % de las infecciones por hongos en pacientes hospitalizados, con una mortalidad asociada del 78 % ^12^.

La disparidad de las cifras en los estudios citados puede explicarse por diferencias en las características demográficas, en la práctica clínica y en las comorbilidades. Sin embargo, la verdadera incidencia de candidiasis invasiva puede ser mayor debido al alto porcentaje de cultivos falsos negativos (30 a 50 %) y a la dificultad del diagnóstico de candidiasis invasiva sin candidemia, como se verá más adelante ^7^.

En los últimos años, se ha observado a nivel mundial un incremento de la frecuencia de una especie emergente: *C. auris.* Este microorganismo se ha detectado en Asia, Europa, Estados Unidos y Suramérica ^18^. En Colombia, se han documentado brotes en la Costa Atlántica y se han observado casos en ciudades andinas ^19^, así como brotes en algunos hospitales ^20^. En el primer brote reportado, hubo una gran mortalidad en los 17 pacientes con infección por esta levadura ^21^. Los factores de riesgo establecidos no parecen diferir mucho de los ya reportados, aunque aún no es claro por qué este microorganismo, previamente desconocido, se ha diseminado a nivel mundial.

La frecuencia de las diferentes especies de *Candida* en Colombia se presenta en el cuadro 2 ^1^^,^^3^^-^^6^^,^^11^^,^^22^. Se observa que los datos tomados en su conjunto reflejan una disminución de la frecuencia relativa de *C. albicans,* con incremento de otras especies, especialmente *C. parapsilopsis.* También, se ha observado un incremento relativo de la frecuencia de *C. glabrata.* No es claro el significado de estos cambios, aunque algunas especies, como *C. parapsilosis,* parecen tener mayor facilidad para colonizar a los trabajadores de la salud y formar biopelículas, lo cual favorecería su transmisión cruzada y podría explicar parcialmente los cambios observados ^13^^,^^14^^,^^23^.

**Cuadro 2:** Distribución de especies de *Candida* en estudios colombianos de pacientes con infección del torrente sanguíneo

Estudio y años de recolección	**Zuluaga-Rodríguez,*et al*. (3) 2001-2007 n (%)**	**Cortés,*et al*. (4) 2001-2007 n (%)**	**Cortés,*et al*. (1) 2008-2009 n (%)**	**Nucci,*et al*. (5) 2008-2010 n (%)**	**Ortiz,*et al*. (11) 2008-2012 n (%)**	**Berrío,*et al*, (6) 2010-2011 n (%)**	**Motoa,*et al*. (22) 2010-2013 n (%)**
*Candida albicans*	147 (43,6)	214 (56)	87 (66,4)	253 (37,6)	42 (52)	60 (40,8)	21 (41,2)
*Candida tropicalis*	79 (23,4)	66 (17,3)	14 (10,6)	118 (17,6)	14 (17)	25 (17)	9 (17,6)
*Candida parapsilosis*	47 (13,9)	61 (16)	19 (14,5)	178 (26,5)	14 (17)	34 (23,1)	7 (13,7)
*Candida glabrata*	32 (9,5)	10 (2,6)	2 (1,5)	42 (6,3)	5 (6)	10 (6,8)	4 (7,9)
*Candida krusei*	11 (3,3)	3 (0,8)	0	18 (2,7)	2 (3)	3 (2,04)	0

La candidemia puede presentarse sobre todo en dos situaciones: usualmente, en unidades de cuidados intensivos en pacientes sin neutropenia críticamente enfermos y con factores predisponentes y, la segunda, en pacientes con enfermedad oncológica y con neutropenia secundaria a la quimioterapia, generalmente por tumores hematológicos ^4^. En los estudios realizados en los hospitales de Colombia, entre 37 y 45 % de los pacientes estaban hospitalizados en unidades de cuidados intensivos y, en una cuarta parte de ellos, el cáncer era el factor de riesgo predominante ^2^.

En otro estudio en el país, se encontró que la candidemia era la quinta causa de infecciones del torrente sanguíneo y la infección fúngica invasiva más común, y que era responsable de cerca del 75 % de todas las micosis invasivas en los hospitales ^12^^,^^24^, con el consecuente aumento de los costos, la morbilidad y la mortalidad, especialmente en las unidades de cuidados intensivos ^25^. Se estima que más del 10 % de las infecciones asociadas con la atención en salud son causadas por *Candida* spp. y que cerca de la mitad de estas ocurren en dichas unidades. La incidencia de la candidiasis invasiva en estas unidades es diez veces mayor que en los servicios quirúrgicos o de hospitalización ^25^. El costo estimado de cada episodio de candidiasis invasiva en adultos hospitalizados es de aproximadamente USD$ 40.000 ^26^ y, en Colombia, los costos totales de atención de pacientes con candidemia equivalieron a COP$ 6.437'210.186 en el 2013 ^27^.

## Diagnóstico

La presentación clínica se caracteriza por síntomas inespecíficos; las manifestaciones más comunes son fiebre, escalofríos y otros signos de reacción inflamatoria que no mejoran con la terapia antibiótica. Si la infección se extiende a otros órganos, por ejemplo, riñones, hígado, huesos, músculos, articulaciones, bazo u ojos, se desarrollan síntomas determinados según el sitio de la infección. Además, el paciente puede presentar falla orgánica.

En un estudio en el país, se estableció que el 83 % de los pacientes presentaba inflamación en el momento de la detección de la candidemia, 25 % tenía disfunción de tres o más órganos y 34 % se encontraba en choque hemodinámico ^1^. Dado el impacto que tiene en el pronóstico de la salud visual, la selección del antifúngico y la duración del tratamiento, es muy importante detectar la infección ocular en los casos de candidemia.

En los pacientes críticamente enfermos, el diagnóstico de candidemia no es fácil, dado que los signos y los síntomas varían drásticamente y que los procedimientos diagnósticos existentes tienen serias limitaciones ^7^.

El diagnóstico se basa en la comprobación mediante métodos microbiológicos, examen directo, histopatología, cultivo de sitios estériles (que requieren procedimientos invasivos) o mediante pruebas serológicas para anticuerpos y antígenos, y moleculares para detectar el ADN de *Candida* spp. ^12^. Los hemocultivos continúan siendo la piedra angular del diagnóstico, sin embargo, su sensibilidad es poca (30 a 50 %) y requieren largos periodos de incubación ^28^. Los nuevos métodos de cultivo tienen mayor capacidad de detección de *Candida* spp. (70 %), pero requieren mínimo de 24 a 48 horas para mostrar positividad, por lo que el resultado puede ser tardío ^29^.

El diagnóstico temprano es necesario, ya que el inicio tardío del tratamiento se asocia con aumento de la mortalidad. Se deben considerar primero los factores de riesgo del paciente, luego valorar las características clínicas y, por último, adelantar los estudios microbiológicos ^29^. Este proceso implica recurrir al tratamiento empírico basado en los factores de riesgo y los índices de predicción de candidiasis. Los más usados en nuestro medio son el índice de colonización de Pittet y el puntaje de *Candida* (o índice de León) ^30^. La utilidad de estos índices está determinada por su valor predictivo negativo, ya que la posibilidad de candidemia es extremadamente baja, con índices negativos en pacientes sin neutropenia ^24^.

Para un diagnóstico más rápido y oportuno, se han desarrollado marcadores serológicos que detectan componentes de la pared celular del hongo, como los manano-oligosacáridos y el 1,3-p-D-glucano. Estos marcadores no se utilizan rutinariamente en nuestro medio. En la candidiasis invasiva, los hemocultivos y la reacción en cadena de la polimerasa (PCR) en pruebas combinadas de antígenos y anticuerpos (manano y anticuerpos antimanano) alcanzan una especificidad del 90 al 100 % y una sensibilidad del 30 al 60 % para la detección de *Candida* spp. ^24^. Las pruebas moleculares son prometedoras, pero aún son de acceso limitado. Es claro que el diagnóstico requiere de los estudios moleculares de laboratorio, y que las técnicas más novedosas para la identificación de los productos de la PCR aportan mayor sensibilidad y especificidad ^31^.

## Pronóstico

La candidiasis invasiva conlleva aumento de la mortalidad en el caso del inicio tardío del tratamiento. La ausencia de métodos de gran sensibilidad y especificidad para el diagnóstico rápido, creó la necesidad de determinar los factores de riesgo y recurrir a métodos de evaluación para garantizar el tratamiento oportuno, incluso en ausencia de evidencia microbiológica de infección ^7^.

La candidemia es un evento terminal en una significativa proporción de pacientes. La tasa de letalidad oscila entre el 40 y el 60 % al mes del episodio y, mayor, en los estudios con seguimiento a un año. La mortalidad a corto plazo sugiere que un episodio de candidemia es un signo ominoso; sin embargo, en ausencia de una enfermedad subyacente grave, la candidemia se considera una condición más benigna ^32^. En un estudio en hospitales de Bogotá, se informó que la tasa de mortalidad durante la hospitalización alcanzó el 36 % ^1^, en tanto que la mortalidad global latinoamericana en un estudio multicéntrico fue de 40 % ^2^. No se encontraron estudios en nuestro medio que permitieran establecer la mortalidad atribuible a candidemia.

El principal factor de mejoría del pronóstico es el inicio temprano del tratamiento antifúngico. Si el tratamiento se inicia en el momento de conocer la positividad de los hemocultivos o durante las primeras 12 horas, la mortalidad es de 10 a 15 % y, si su inicio se retrasa 48 horas, la mortalidad asciende a 30 a 35 % ^33^.

Otro factor asociado con un mejor pronóstico es el retiro temprano del catéter venoso central. Las guías aconsejan que, en los pacientes con candidemia, se retire tan pronto como sea posible ^26^^,^^34^, pues, aunque el acceso venoso no siempre es el origen de la infección, es un reservorio que prolonga la candidemia e incrementa el riesgo de focos metastásicos de infección, por lo que su retiro se asocia con una menor duración y mortalidad por la infección ^35^.

Otra acción relevante para mejorar el pronóstico es la exploración ocular. Todas las guías recomiendan hacer una exploración del fondo del ojo, dado que se ha reportado la incidencia de endoftalmitis o coriorretinitis por *Candida* spp. en 5 a 78 % de los pacientes con candidemia ^36^.

### Tratamiento de la candidemia en pacientes sin neutropenia

La candidemia requiere tratamiento antifúngico durante varias semanas; su tipo y su duración dependen de factores específicos del paciente, como la edad, las comorbilidades, el estado inmunológico y la gravedad de la infección, así como la sensibilidad de la especie identificada ^37^. Hay diferentes antifúngicos disponibles para el tratamiento de la candidiasis (anfotericina B, flucitosina, azoles), sin embargo, con este grupo de medicamentos se han documentado efectos adversos importantes debidos a su toxicidad o a las interacciones farmacológicas. Más recientemente, se han incorporado las equinocandinas (anidulafungina, caspofungina y micafungina), fungicidas que tienen un amplio espectro de acción con mayores tasas de éxito clínico y son bien tolerados ^38^.

Es importante resaltar que, entre dichos medicamentos, hay diferencias importantes en el perfil de uso clínico, derivadas de sus características farmacocinéticas particulares (cuadro 3).

**Cuadro 3:** Diferencias de los efectos adversos e interacciones farmacológicas de las diferentes equinocandinas

Caspofungina	Micafungina	Anidulafungina
Interacciones con rifampicina, fenitoína, carbamazepina, antirretrovirales y dexametasona	Ausencia de evidencia de eficacia y seguridad en falla hepática grave	No tiene ningún grado de metabolismo hepático o renal.
Requiere ajuste de la dosis en pacientes con falla hepática (clasificados como Child-Pough B); se desconoce la dosis adecuada en falla hepática grave.	El reporte de formación de tumores hepáticos en roedores genera preocupación.	No requiere ajuste de dosis en falla renal o hepática.

Adaptado de: Paramythiotou E, *et al.* Invasive fungal infections in the ICU: how to approach, how to treat. Molecules. 2014;19:1085-119.

En el cuadro 4 se presentan esquemas terapéuticos recomendados para el manejo de la candidiasis invasiva o candidemia en cuatro situaciones, todos ellos formulados en los últimos seis años, siendo la más reciente actualización la de la guía de la *Infectious Diseases Society of America* (IDSA).

**Cuadro 4:** Medicamentos disponibles o recomendados para diferentes situaciones de candidemia

Cuadro clínico	Medicamento de elección	Medicamentos alternativos
Candidemia	Caspofungina o anidulafungina	Anfotericina B liposómico Anfotericina B deoxicolato Fluconazol Voriconazol
Candidemia por C. *parapsilosis*	Fluconazol	Voriconazol Anfotericina B liposómico Anfotericina B deoxicolato
Candidemia con compromiso ocular	Fluconazol	Voriconazol
Candidemia con compromiso del sistema nervioso central	Voriconazol	Anfotericina B deoxicolato

Reproducido de: Cortés JA, Prada G. Protocolo de estudio y manejo de pacientes con candidiasis sistémica en adultos. Infectio. 2012;16(Supl 3):118-22 24.

El actual consenso colombiano de práctica clínica para el manejo de la candidemia es una adaptación de las guías internacionales, especialmente, la estadounidense y la europea ^39^. También, se cuenta con el protocolo de estudio y manejo de pacientes con candidiasis sistémica de la Asociación Colombiana de Infectología (ACIN) ^24^, el cual se resume en el cuadro 5.

**Cuadro 5:** Dosis recomendadas y efectos adversos de medicamentos utilizados en el manejo de la candidemia

Medicamento	Dosis	Efectos adversos frecuentes	Observaciones
Deoxicolato de anfotericina B	0,7-1 mg/kg/día	Falla renal, hipopotasemia, fiebre y otros efectos de la infusión	La anfotericina se debe acompañar de una hidratación apropiada antes de la infusión y de acetaminofén o meperidina como medicación previa para prevenir reacciones a la infusión. La infusión es prolongada y se puede hacer de forma continua. Requiere monitorización de la función renal y del nivel de potasio.
Anfotericina B liposómica	3-5 mg/kg/día	Falla renal con menor frecuencia, disnea y otros efectos de la infusión	La infusión inicial es de 2 h, y puede acortarse en pacientes que lo toleren. Requiere monitorización de la función renal y los niveles de potasio.
Anfotericina B complejo lipídico	5 mg/kg/día	Falla renal menos frecuente, disnea y otros efectos de la infusión	Su uso está limitado a pacientes intolerantes al deoxicolato de anfotericina B
Anidulafungina	100 mg/día, dosis inicial de 200 mg	Limitados	Menor interacción medicamentosa con rifampicina, carbamazepina e inmunosupresores
Caspofungina	50 mg/día, dosis inicial de 70 mg	Limitados	Interacciones medicamentosas diversas (ciclosporina, carbamazepina, rifampicina y otros); no usar en falla hepática grave
Micafungina	100 mg/día	Dolor abdominal, náuseas, diarrea	
Fluconazol	400 mg/día, dosis inicial de 800 mg	Elevación de transaminasas	Requiere ajuste a la función renal
Voriconazol	4 mg/kg cada 12 h, dosis inicial de 6 mg/kg en 2 dosis para personas de >40 kg	Elevación de transaminasas, cambios en la visión de colores	Niveles variables, especialmente con administración oral

Reproducido de: Cortés JA, Prada G. Protocolo de estudio y manejo de pacientes con candidiasis sistémica en adultos. Infectio. 2012;16(Supl 3):118-22.

Este protocolo y las guías de manejo privilegian el uso de las equinocandinas (anidulafungina, micafungina y caspofungina), sin que se haya documentado una mayor efectividad de una sobre las otras y con diferencias importantes en la farmacocinética ^40^. Se destaca que la anidulafungina tiene un menor riesgo de interacciones farmacológicas. El tratamiento se debe adecuar según los patrones de sensibilidad obtenidos con los cultivos ^38^.

Las guías europeas de manejo ^29^ recomiendan las equinocandinas como primera línea de tratamiento de la candidemia en pacientes sin neutropenia y plantean que hay menos datos en favor del fluconazol. Estas guías destacan el bajo riesgo de interacciones de la anidulafungina comparada con la caspofungina. Se ha observado que las equinocandinas tienen efecto sobre las especies de *Candida* en biopelículas ^41^, lo cual podría tener un impacto en el tratamiento de las infecciones relacionadas con dispositivos o con la endocarditis. También, se han observado diferencias entre las equinocandinas en las biopelículas según la especie involucrada ^42^.

En general, en todas las guías (cuadro 4) se proponen las equinocandinas como tratamiento de primera línea de la candidemia y se considera la anidulafungina una opción válida para el manejo pacientes neutropénicos y sin neutropenia ^2^, tal como se ha recomendado también en Brasil ^43^.

En un análisis de costo-efectividad en el Reino Unido basado en un metaanálisis sobre la candidemia, en el cual se comparó la anidulafungina con la caspofungina, la micafungina y el fluconazol, se observó una mayor tasa de mejoría con todas las equinocandinas comparadas con el fluconazol. La tasa de supervivencia con anidulafungina fue mayor comparada con la de todos los demás medicamentos ^44^. En otro metaanálisis, también se demostró la superioridad de las equinocandinas ^45^.

La interpretación de los estudios científicos sobre el tema es compleja debido a que el número de estudios clínicos disponibles es pequeño y no incluyen todas las posibles comparaciones directas ^46^^-^^48^. Se estima que la anfotericina y las equinocandinas tienen una eficacia similar, con un mejor perfil de seguridad de las equinocandinas. El problema más importante de la anfotericina, aunque no el único, es la gran frecuencia de falla renal aguda asociada con el tratamiento. Las formas lipídicas de la anfotericina, especialmente la liposómica, han disminuido la frecuencia de este problema ^49^. De todas maneras, se recomienda abstenerse de usar las anfotericinas en pacientes con alto riesgo de falla renal o falla renal establecida ^50^.

Dicho esto, es importante recordar las situaciones en las que los azoles son mejores que las equinocandinas, incluidos los casos con compromiso ocular o del sistema nervioso central (cuadro 4). Hay controversia sobre el tratamiento de las infecciones por *C. parapsilopsis* con las equinocandinas, debido a que su concentración inhibitoria mínima es más elevada en comparación con la encontrada con otras especies de *Candida.* Sin embargo, en los estudios clínicos no se ha determinado una diferencia estadísticamente significativa a favor de ninguno de los productos ^51^. Tampoco es claro si hay diferencias en la eficacia de las diversas equinocandinas debidas a su farmacocinética ^40^; el beneficio potencial podría darse en la selección de cepas resistentes y las interacciones medicamentosas. La frecuencia de descontinuación fue más elevada con la caspofungina, aunque mucho menor comparada con la anfotericina B (3,8 % *Vs.* más de 10 %) ^52^.

## Resistencia a los antifúngicos

Se han observado diferentes tasas de resistencia a los antimicóticos en el país. Las variaciones en la resistencia al fluconazol se deben fundamentalmente al tipo de técnica utilizada y a los pacientes observados. Las tasas de resistencia también varían según la especie identificada, ya que *C. krusei* es intrínsecamente resistente al fluconazol y *C. glabrata* tiene sensibilidad disminuida frente a este ^1^^,^^53^. En el estudio latinoamericano citado, se utilizaron técnicas de referencia (dilución en caldo) y es la muestra de aislamientos más grande de la región. En este estudio, la resistencia al fluconazol y a las equinocandinas fue muy baja ^2^. Sin embargo, se han identificado aislamientos con mutaciones de resistencia en nuestro medio ^54^.

## Conclusiones

En Colombia, la candidemia es una entidad relativamente frecuente, con tasas de incidencia y prevalencia más altas que en otras partes del mundo; es la quinta causa de infección del torrente sanguíneo en las unidades de cuidados intensivos y representa el 88 % de las infecciones fúngicas en pacientes hospitalizados, con una mortalidad que oscila entre el 36 y el 40 %.

El diagnóstico de la candidiasis debe basarse en la identificación de grupos de riesgo con mayor frecuencia de la infección. Los hemocultivos permiten la identificación de la especie y la posibilidad de establecer la sensibilidad antifúngica, sin embargo, están limitados por su poca sensibilidad. Las nuevas estrategias incluyen técnicas moleculares que deben implementarse ampliamente para mejorar el diagnóstico, disminuir la mortalidad y reducir el uso incorrecto de los antifúngicos.

El principal factor de mejoría del pronóstico es el inicio temprano del tratamiento antifúngico ^33^. Las guías desarrolladas con base en revisiones sistemáticas de la literatura recomiendan el uso de las equinoandinas en los pacientes inestables. Otras alternativas incluyen el fluconazol y la anfotericina B, aunque con limitaciones en la eficacia y la seguridad, respectivamente.
